# Indiana Association of Veterinary Graduates

**Published:** 1893-11

**Authors:** 


### INDIANA ASSOCIATION OF VETERINARY GRADUATES.

The semi-annual meeting of the Indiana Association of Veterinary Graduates was held August 21st and 22nd, 1893, at Newcastle, Ind.

*First Day's Session, August 21st.*

In the absence of the President, the Vice-President, C. F. Bell, occupied the chair; Dr. J. E. Cloud, Secretary. On roll call Drs. C. F. Bell, F. A. Balsee, Lee Hoover, F. Braginton, C. M. Stull, W. B. Wallace, J. C. Rodgers, O. G. Whitestine, and J. E. Cloud responded.

The minutes of the previous meeting were read and approved. The reports of the Secretary and of the Treasurer were read and acccepted.

*J. H. Mahoney*, proposed by L. Hoover and seconded by J. C. Rodgers was elected a member.

*Dr. Lee Hoover* read a communication on Omphalo-Phlebitis. He defined the disease as a functional trouble occuring soon after birth, common in breeding districts, and affecting usually the equine, bovine and ovine species, but seen in the canine and porcine; sometimes almost enzoötic. He followed in general the etiology of Bollinger, stating that "It is not essentially due to a specific bacteria, but may be and often is caused by the products of decomposition of nitiogenous matter, without the presence of living micro-organisms." He impressed the necessity of prompt and vigorous prophylactic treatment, recommending absolute cleanliness, and the use of Iodoform, Boracic Acid and surgical collodion. For remedial treatment he stated that salicylate of soda should be alternated with preparations of carbolic acid, sulphate and hyposulphite of soda. Diffusive stimulants and laxatives might be needed. He recommended surgical interference only in abscesses near the joints. For anodyne lotion the author uses a mixture of arnica, witch hazel, and laudanum ; for cooling lotion equal parts of muriate of ammonia and nitrate of potash, to 16 parts of water.

*Dr. C. M. Stull* furnished a valuable paper on Veterinary Legislation which was ordered printed and a copy sent to each veterinary graduate in Indiana.

*Drs. Bell, Balser and Stull* were appointed a Committee on Legislation.

*Drs. Stull, Rodgers and Hoover* a Committee on the " Indiana Veterinary College " reported as follows :

WHEREAS, We have situated in the city of Indianapolis, State of Indiana, an institution called the Indiana Veterinary College, and the prospectus issued by said institution calling for three sessions of thirteen weeks each, and allowing an empiric to become qualified in one session at the said institution, therefore be it

*Rosolved,* That we as a body, representing the Indiana Association of Veterinary Graduates, do denounce an institution called the Indiana Veterinary College, situated in the city of Indianapolis, State of Indiana, also graduates and honorary graduates of said institute.

*Resolved,* That we as a body humbly beseech your honorable body to give this due consideration, as we deem it an unfit institute for obtaining a veterinary education.

The report of the Committee on the Revision of the Constitution and By-Laws was accepted as presented by the committee.

*Committee on Programme,* J. C. Rodgers, C. M. Stull and J. E. Cloud.

*Committee on Arrangements,* F. A. Balser, J. C. Wallace, C. F. Bell.

Adjourned to meet on the morning of the 22d.

Dr. Balser assisited by Dr. Whitestine, successfully performed a very difficult and interesting operation for a fistulous opening of the œsophagus, which added a practical lesson for the day.

*Second Day's Session, August 22d.*

*Drs. Stull, Balser and Cloud* were appointed delegates to the United States Veterinary Congress to be held in Chicago.

On motion, the rules were suspended and a board of censors was appointed, consisting of Drs. Mahoney, Balser and Wallace.

It was moved and adopted that the Association shall procure a charter. The Secretary was ordered to obtain same and have it properly recorded.

*Dr. Galbraith,* of Macon, Georgia, was elected an associate member.

A bill presented by Secretary for printing programmes was approved.

On motion the meeting adjourned to meet at Fort Wayne, on December 6th and 7th, 1893.

